# PDX1 Methylation, NGN3 and Pax6 Expression Levels in Pregnant Women with Gestational Diabetes Mellitus and their association with neonatal blood sugars and birth weight

**DOI:** 10.12669/pjms.40.3.7700

**Published:** 2024

**Authors:** Yuanyuan Su, Jingjing Li, Limin Chen, Hongnan Lian, Yamin Qie

**Affiliations:** 1 Yuanyuan Sum, Department of Obstetrics and Gynecology Affiliated Hospital of Hebei University, Baoding 071000, Hebei, China; 2 Jingjing Li, Department of Obstetrics and Gynecology Affiliated Hospital of Hebei University, Baoding 071000, Hebei, China; 3 Limin Chen, Department of Obstetrics and Gynecology Affiliated Hospital of Hebei University, Baoding 071000, Hebei, China; 4 Hongnan Lian Department of Obstetrics and Gynecology Baoding Maternal and Child Health Hospital, Baoding 071000, Hebei, China; 5 Yamin Qie Department of Obstetrics and Gynecology, Wangdu County Hospital of Traditional Chinese Medicine, Baoding 071000, Hebei, China

**Keywords:** Gestational diabetes mellitus, PDX1 methylation, NGN3, Pax6, Neonatal blood glucose

## Abstract

**Objective:**

To investigate the correlation of maternal PDX1 methylation, NGN3 and Pax6 expression levels with neonatal blood sugars and birth weight in pregnant women with GDM and non GDM.

**Methods:**

This was a prospective cohort study. Total 80 pregnant women who were examined and delivered in the Department of Obstetrics of Affiliated Hospital of Hebei University from January 2019 to June 2022 were recruited and divided into two groups according to the results of oral glucose tolerance test (OGTT): the control group and the observation group, with 40 cases in each group. PDXl methylation rate was measured by the methylation-specific PCR method, whereas gene expression levels of PDX1, NGN3 and Pax6 were measured by RT-PCR meanwhile, neonatal blood glucose and hemoglobin A1c (HbA1c) levels were also measured.

**Results:**

In comparison with the control group, the observation group had higher levels of FBG, 2-hour postprandial blood glucose (2hPBG) and HbA1c (P<0.05). Neonatal birth weight and insulin levels in the observation group were significantly higher than those in the control group, while Apgar scores and blood glucose were lower than those in the control group(P<0.05). Moreover, the observation group had significantly lower gene expression levels of PDX1, NGN3 and Pax6, and a higher PDX1 methylation rate than the control group (P<0.05). Correlation analysis revealed a negative correlation between neonatal blood glucose levels and PDX1, NGN3 and Pax6 levels in the observation group, with statistical significance (P<0.05).

**Conclusion:**

Changes in maternal PDX1 methylation, NGN3 and Pax6 expression levels may lead to abnormal glucose metabolism in neonates, which has a close bearing on neonatal hypoglycemia and blood glucose levels caused by GDM.

## INTRODUCTION

During pregnancy, a series of changes occur in the endocrine system of pregnant women, resulting in certain changes in the metabolism of sugar, fat and protein, etc. This abnormality in glucose metabolism that occurs during pregnancy is commonly referred to as gestational diabetes mellitus (GDM). GDM is one of the common complications of pregnancy in obstetrics and gynecology.^1^

Most patients with GDM will have their blood glucose levels return to normal gradually after delivery, but some will struggle to return to normal after delivery and may even progress to type-2 diabetes mellitus(T2DM).^2^ GDM, with a current trend of increasing incidence year by year, poses a great danger and risk to the pregnant woman and the fetus, and significantly increases the probability of diabetes mellitus and metabolic syndrome after delivery.^3^ Studies have revealed many adverse effects of GDM on pregnancy outcomes and neonates, including pregnancy-induced hypertension, premature rupture of membranes, macrosomia, fetal respiratory distress syndrome, and neonatal hypoglycemia.^4^,^5^ Among them, the occurrence of neonatal glucose metabolism disorder is closely related to GDM, with a mechanism of occurrence that is not yet clear. PDX1 (pancreatic and duodenal homeobox-1), a specific transcription factor on human chromosome 13q12.1, plays a crucial role in regulating the function of pancreatic islet β cells. Alternatively, methylation of PDX1 leads to permanent silencing of its gene.

A study^6^ showed that decreased expression of genetic and acquired PDX1 can lead to T2DM and islet β-cell dysfunction. NGN3 (Neurogenin-3), a member of the transcription factor family, is a determinant gene for pancreatic endocrine development. In case of reduced expression of NGN3, it leads to dysfunction of pancreatic endocrine. Pax6 (paired box gene6) plays a crucial role in the development of pancreatic endocrine cells. Although the correlation between PDX1, NGN3 and Pax6 gene expression and GDM has been reported,^7^,^8^ there are few clinical reports on the relationship between PDX1 gene methylation, NGN3 and Pax6 expression levels in pregnant women with GDM and neonatal glucose levels. In this study, PDX1 gene methylation rate, NGN3 and Pax6 expression levels in pregnant women with GDM were analyzed to investigate their relationship with neonatal blood glucose. The necessity of early diagnosis and treatment of gestational diabetes was emphasized in order to improve the abnormal blood sugar and birth weight of newborn.

## METHODS

This was a prospective cohort study. Eighty pregnant women who were examined and delivered in the Department of Obstetrics of Affiliated Hospital of Hebei University from January 2019 to June 2022 were recruited, all of whom completed OGTT and PDX1 methylation, NGN3 and Pax6 expression levels assessment during pregnancy. The diagnosis of GDM was in accordance with the diagnostic criteria of the Chinese Guidelines for Diagnosis and Treatment of Gestational Diabetes Mellitus (2014)^9^ developed by the Department of Obstetrics and Gynecology, the Obstetrics and Gynecology Branch of the Chinese Medical Association, according to which 80 pregnant women were divided into two groups: the control group (OGTT normal group) and the observation group (GDM group), with 40 cases in each group. Pregnant women with hypertension, use of drugs affecting glucose metabolism, and liver and kidney disease were excluded.

### Ethical Approval:

This study was approved by the ethics committee of Affiliated Hospital of Hebei University (No.: HDFYLL-KY-2023-052; date: March 13,2023), and the families of neonates signed written informed consent.

### Data and specimen collection:

The age, gestational week, gravidity and BMI of all pregnant women were collected. Before delivery, 10 ml of elbow venous blood was drawn from pregnant women on an empty stomach, part of which was used to measure biochemical indicators and the other part was used for the extraction of peripheral blood mononuclear cells. Subsequently, the mononuclear cells were processed according to the procedure, and the extracted lysed DNA was stored in a -40°C refrigerator for backup. Immediately after delivery, the neonates were weighed and the neonatal Apgar score (one minutes, five minutes) was recorded.

### Measurement of biochemical indicators:

Venous blood was drawn from neonates within 30 min after birth, and neonatal insulin levels were measured by radioimmunoassay. Furthermore, blood glucose levels were measured by glucose oxidase method, whereas hemoglobin A1c (HbA1c) levels were measured by agarose gel electrophoresis with automatic glycated hemoglobin analyzer.

Measurement of PDX1 gene methylation rate: Measurement by methylation-specific PCR method: The DNA concentration was measured after DNA purification of peripheral blood mononuclear cell (PBMCs), and individual genomic DNA was transformed using the heavy sulfite method after the ideal concentration was reached. PDX1 gene fragments were amplified by hot-start PCR using the transformed DNA as a template. In terms of PDX1 primers, they were forward: 5’-GGCACAGCGGAGCCTAT-3’ and reverse: 5’-GCCACCC-CTGCAGCTT-3’ with a fragment length of 354 bp. With a total volume of 25μl of the reaction system, PCR was amplified under the conditions of pre-denaturation at 95°C for two minutes, denaturation at 94°C for 20s, annealing at 54°C for 30s, and extension at 67°C for 20s, for a total of 40 cycles.

**Fig.1:**
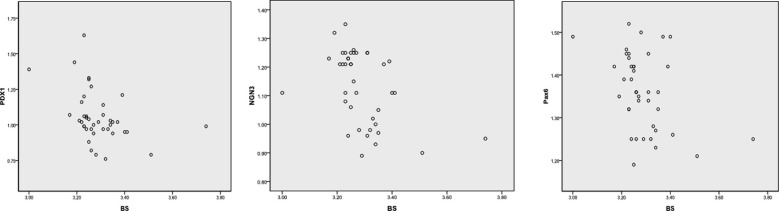
Correlation analysis of neonatal blood glucose levels with maternal PDX1, NGN3 and Pax6 levels.

After the cycle, the PCR was extended at 72°C for five minutes. The purified fragments were connected with PMD18-T vector, and 10 single positive clones were selected from each group of samples after transformation and coating for sequencing, which was repeated for three times. If the cyclic threshold was 37 or below, the fluorescence intensity at the end point of amplification was 2-3 times that of the negative control group, and the dissolution curve was unimodal and the corresponding temperature was consistent with the so-called temperature of the positive control, it was determined to be positive, and methylation would be determined after three consecutive positive times. Methylation rate = number of methylated patients/total number of patients.

Measurement of mRNA expression levels of NGN3 and Pax6: RT-PCR was employed to measure the mRNA expression levels of NGN3 and Pax6, Trizol was utilized to extract total RNA from peripheral blood mononuclear cells for reverse transcription, and NGN3 and Pax6mRNA were amplified by PCR.

### Statistical Analysis:

All data in this study were statistically analyzed using SPSS22.0 software. Quantitative data were expressed as mean ± standard deviation (*x̅*±S), and *t* test was used for comparison between the two groups before and after treatment. Qualitative data was expressed as *n* (%), and *χ*^2^ test was used for comparison between the two groups. Correlation analysis between variables was performed using Pearson’s linear correlation. *P*<0.05 indicates a statistically significant difference.

## RESULTS

No statistically significant differences were observed between the two groups in terms of mean age, gestational age at delivery and BMI (P>0.05). Fasting blood glucose (FBG), 2h postprandial blood glucose(2hPBG) and HbA1c were higher in the observation group than in the control group, with statistically significant differences(P<0.05), Table-I.

**Table-I:** Comparison of general data between the two groups of pregnant women (*χ̅*±S).

Group	Age (years)	Gestational age of delivery (weeks)	FBG (mmol/L)	2hPBG (mmol/L)	HbA1c (%)	BMI (kg/m^2^)
Observation group	30.75±3.22	39.63±1.58	7.09±0.33	8.52±0.38	6.38±0.81	26.13±2.99
Control group	30.10±2.77	39.18±1.48	4.92±0.64	6.36±0.65	4.73±0.60	25.48±1.55
*t* value	0.968	1.313	19.114	18.252	10.4149	1.221
*P* value	0.336	0.193	0.000	0.000	0.000	0.226

The observation group had significantly higher neonatal birth weight and insulin level, but lower Apgar score and blood glucose than the control group, with statistically significant differences(P<0.05), Table-II.

**Table-II:** Comparison of neonatal conditions between the two groups (*χ̅*±S).

Group	Birth weight1(g)	1minute Apgar score(points)	5min Apgar score	Insulin(mU/L)	Blood glucose(mmol/L)
Observation group	3621.32±90.08	8.48±0.78	9.28±0.68	10.16±1.23	3.29±0.11
Control group	3328.25±82.77	9.03±0.73	9.68±0.47	7.69±1.30	3.95±0.13
t value	15.152	3.240	3.055	8.724	24.267
P value	0.000	0.002	0.003	0.000	0.000

The gene expression levels of PDX1, NGN3 and Pax6 in the observation group were significantly lower than those in the control group, with statistically significant differences (P<0.05). In addition, the PDX1 methylation rate was higher in the observation group compared with the control group, with a statistically significant difference (P<0.05), Table-III. Neonatal blood glucose levels in the observation group were negatively correlated with PDX1, NGN3 and Pax6 levels, with statistically significant correlation (P<0.05). See Table-IV.

**Table-III:** Comparison of gene expression levels of PDX1, NGN3 and Pax6 and PDX1 methylation rate between the two groups (*χ̅*±S).

Group	NGN3 expression	Pax6 expression	PDX1	

			Expression	Methylation rate
Observation group	1.13±0.13	1.36±0.09	1.06±0.18	35(87.50)
Control group	1.22±018	1.43±0.13	1.35±0.16	25(62.50)
*t* value	2.612	2.653	7.695	6.667
*P* value	0.011	0.010	0.000	0.010

**Table-IV:** Correlation analysis of neonatal blood glucose levels with PDX1, NGN3 and Pax6.

	PDX1	NGN3	Pax6
*r*	-0.390	-0.447	-0.397
*P*	0.013	0.004	0.011

## DISCUSSION

The results of this study suggest that neonatal hypoglycemia may be related to the decreased expression levels of PDX1, NGN3 and Pax6 genes in pregnant women with GDM. A study^10^ revealed an association between the development of T2DM and pancreatic β cell dysfunction and decreased genetic or acquired PDX1 expression in human and animal models. Another study has shown that decreased expression of PDX1 gene has a close bearing on the occurrence of GDM and neonatal birth weight change.^11^,^12^ As shown in the results of this study, the relative expression of PDX1 in the GDM group was lower than that in the control group (P<0.05), and the gene methylation rate was higher than that in the control group (P<0.05), suggesting a correlation between the decreased expression of PDX1 gene and the increased methylation level and the occurrence of GDM.

A study^13^ showed that NGN3 is expressed in endocrine progenitor cells but not in pancreatic progenitor cell phase, so it is believed to play a regulatory role in pancreatic development. It was confirmed in the study of Jiang et al.^14^ that the expression of NGN3 decreased during the development of rat embryonic pancreas and the repair of adult islets, as well as the expression of several downstream genes regulating the differentiation and maturation of islet cells, such as Nkx2.2, NeuroDl and Pax6, resulting in the endocrine dysfunction of pancreatic islets. This was followed by the study of Wang et al.^15^, which showed that other downstream transcription factors of NGN3 and PDX1 could be activated during the development of pancreatic endocrine cells, and they had a synergistic effect. Additionally, Oiver et al.^16^ demonstrated that when Pdxl switches on NGN3, cross networks of downstream transcription factors are also activated, resulting in the directed differentiation of pancreatic progenitor cells or embryonic stem cells into islet β cells. In this study that the expression level of NGN3 was lower in the GDM group than in the control group (P<0.05), which was consistent with the above research conclusions.

The PDX1 gene is located at 13q12.1 and contains an open reading frame encoding 283 amino acids. The region of exon-1 at the 5’ end of the PDX1 gene and the proximal promoter contain highly conserved CpG islands that are important for PDX1. As demonstrated in related studies, PDX1, as an important transcription factor affecting pancreatic directed differentiation and maturation^17^, is required for both pancreatic bud formation during embryonic period and pancreatic β-cell differentiation and function at later stages. PDX1 binds directly to the insulin gene promoter region and acts as an agonist of the insulin gene. PDX1 is a well-established tumor suppressor gene with down-regulated expression in patients with gastric cancer.^18^

However, less attention has been given as of now to the association of PDX1 gene methylation with GDM and its pregnancy outcome and neonatal glycemia. The Pax6 gene, located on human chromosome 11, is composed of 13 introns and 14 exons and plays an important role in pancreatic development. It was confirmed in a study^19^ that Pax6 gene mutation would lead to reduced expression of the hormone converting enzyme PC1/3, which blockes the conversion of pre-insulin to insulin. As a result, insulin sensitivity is reduced and glucose tolerance is abnormal, resulting in a drop in blood glucose concentration. Other studies have shown that Pax6 is also involved in the transcriptional regulation of islet specific glucose six phosphatase catalytic subunits^20^, further demonstrating the importance of Pax6 in the regulation of glucose metabolism. Pax6 has been considered as a candidate gene for diabetes due to its role in transcriptional regulation of β-cell insulin secretion. The above studies indicated that Pax6 could regulate insulin transcription and expression, affect insulin and hyperglycemic secretion, and play a key role in impaired glucose metabolism and the occurrence and development of diabetes. The study conclusion of Zhang et al. also showed that Pax6 expression level was negatively correlated with blood glucose^21^, which was consistent with the conclusion of this study. In addition, it was shown in this study that the blood glucose and neonatal insulin levels of the corresponding pregnant women were higher than those in the control group (P<0.05), while the neonatal blood glucose levels were lower than that in the control group (P<0.05), suggesting that NGN3 and Pax6 affected the blood glucose and related factors by affecting the pancreatic β cells.

### Limitations of the study:

There are some limitations in this study. The study was observational. In future, we will focus on genetic testing to diagnose GDM early and improve newborn blood sugar and body weight.

## CONCLUSIONS

The blood glucose level of neonates delivered by GDM patients is lower than that of non-GDM patients, with increased methylation of maternal PDX1 gene and decreased expression levels of maternal PDX1, NGN3 and Pax6 genes, which is related to changes in neonatal blood glucose levels.

### Authors’ Contributions:

**YS** and **LC:** Carried out the studies, participated in collecting data, and drafted the manuscript, and are responsible and accountable for the accuracy or integrity of the work.

**JL** and **HL:** Performed the statistical analysis and participated in its design.

**YQ:** Participated in acquisition, analysis, interpretation of data and draft the manuscript.

All authors read and approved the final manuscript.
